# Biotinylated Surfome Profiling Identifies Potential Biomarkers for Diagnosis and Therapy of Aspergillus fumigatus Infection

**DOI:** 10.1128/mSphere.00535-20

**Published:** 2020-08-12

**Authors:** Lei-Jie Jia, Thomas Krüger, Matthew G. Blango, Ferdinand von Eggeling, Olaf Kniemeyer, Axel A. Brakhage

**Affiliations:** a Department of Molecular and Applied Microbiology, Leibniz Institute for Natural Product Research and Infection Biology—Hans Knöll Institute, Jena, Germany; b Jena University Hospital, Department of Otolaryngology, Jena, Germany; c Jena University Hospital, Core Unit Proteome Analysis, Jena, Germany; d Jena University Hospital, DFG Core Unit Jena Biophotonic and Imaging Laboratory (JBIL), Jena, Germany; e Department of Microbiology and Molecular Biology, Institute of Microbiology, Friedrich Schiller University, Jena, Germany; University College Dublin, Belfield

**Keywords:** *Aspergillus fumigatus*, surface biotinylation, surfome, LC-MS/MS, allergens, heat shock protein, protein chaperone, proteomics, spores, surface proteins

## Abstract

Aspergillus fumigatus is one of the most common airborne molds capable of causing mycoses and allergies in humans. During infection, fungal surface proteins mediate the first contact with the human immune system to evade immune responses or to induce hypersensitivity. Several methods have been established for surface proteomics (surfomics). Biotinylation coupled with liquid chromatography-tandem mass spectrometry (LC-MS/MS) identification of peptides is a particularly efficient method to identify the surface-exposed regions of proteins that potentially mediate interaction with the host. After biotinylation of surface proteins during spore germination, we detected 231 different biotinylated surface proteins (including several well-known proteins such as RodA, CcpA, and DppV; allergens; and heat shock proteins [HSPs]), as well as some previously undescribed surface proteins. The dynamic change of the surface proteome was illustrated by detection of a relatively high number of proteins exclusively at one developmental stage. Using immunofluorescence microscopy, we confirmed the surface localization of several HSPs of the HSP70 family, which may have moonlighting functions. Collectively, by comparing our data with data representative of previously published A. fumigatus surface proteomes, our study generated a comprehensive data set corresponding to the A. fumigatus surfome and uncovered the surface-exposed regions of many proteins on the surface of conidia or hyphae. These surface-exposed regions are candidates for direct interaction with host cells and may represent antigenic epitopes that either induce protective immune responses or mediate immune evasion. Thus, our data sets provided and compiled here represent reasonable immunotherapy and diagnostic targets for future investigations.

**IMPORTANCE** Aspergillus fumigatus is the most important airborne human-pathogenic mold, capable of causing both life-threatening invasive pulmonary aspergillosis in immunocompromised patients and allergy-inducing infections in individuals with atopic allergy. Despite its obvious medical relevance, timely diagnosis and efficient antifungal treatment of A. fumigatus infection remain major challenges. Proteins on the surface of conidia (asexually produced spores) and mycelium directly mediate host-pathogen interaction and also may serve as targets for diagnosis and immunotherapy. However, the similarity of protein sequences between A. fumigatus and other organisms, sometimes even including the human host, makes selection of targets for immunological-based studies difficult. Here, using surface protein biotinylation coupled with LC-MS/MS analysis, we identified hundreds of A. fumigatus surface proteins with exposed regions, further defining putative targets for possible diagnostic and immunotherapeutic design.

## Abstract (precis)

Aspergillus fumigatus is the most important airborne human-pathogenic mold, capable of causing both life-threatening invasive pulmonary aspergillosis in immunocompromised patients and allergy-inducing infections in individuals with atopic allergy. Despite its obvious medical relevance, timely diagnosis and efficient antifungal treatment of A. fumigatus infection remain major challenges. Proteins on the surface of conidia (asexually produced spores) and mycelium directly mediate host-pathogen interaction and also may serve as targets for diagnosis and immunotherapy. However, the similarity of protein sequences between A. fumigatus and other organisms, sometimes even including the human host, makes selection of targets for immunological-based studies difficult. Here, using surface protein biotinylation coupled with LC-MS/MS analysis, we identified hundreds of A. fumigatus surface proteins with exposed regions, further defining putative targets for possible diagnostic and immunotherapeutic design.

## INTRODUCTION

The saprotrophic fungus Aspergillus fumigatus, which occurs on decaying organic material, is associated with a wide spectrum of diseases in humans (1, 2). The inhalation of A. fumigatus airborne conidia may cause life-threatening invasive pulmonary aspergillosis in immunocompromised patients, chronic pulmonary aspergillosis in immunocompetent people with underlying lung diseases, or allergic infections such as allergic bronchopulmonary aspergillosis (ABPA) in individuals with atopic allergy (3, 4). Despite continuous research and improvements of diagnostic tools, timely diagnosis of A. fumigatus infections remains a challenge (1). Detection kits for a few different recombinant allergens of A. fumigatus are now commercially available for the diagnosis of ABPA, but cross-reactivity with antigens from other microorganisms still makes diagnosis difficult (5). In addition to DNA and cell wall polysaccharides, fungal proteins exposed to the surface may serve as candidate diagnostic markers and valuable targets for new therapeutics (6, 7).

The A. fumigatus cell wall not only maintains cellular integrity and protects the cell from external aggression but also serves as a harbor for virulence factors that contribute to immune evasion, adherence, and virulence (8). Although the cell wall is composed of >90% polysaccharides (1), hundreds of different surface proteins have been detected across various proteome studies (9–11). Only a few of these proteins are well characterized, including their roles in A. fumigatus virulence. RodA, which forms the hydrophobic rodlet layer on dormant conidia, is the best-studied conidial surface protein. RodA is immunologically inert and can mask dectin-1- and dectin-2-dependent host responses (12, 13). Although Thau et al. showed that Δ*rodA* conidia remained pathogenic in mice, it was also shown that the altered conidial surface of this strain decreases conidial survival and exacerbates the host immune response during interaction with innate immune cells (9, 12, 14, 15). Another abundant conidial surface protein, CcpA, presumably plays a role in maintaining the spore surface structure and preventing immune recognition. Consequently, it was previously shown to be essential for virulence in a corticosteroid immunosuppressed mouse infection model (10). The A. fumigatus protein CalA, which is present on swollen conidia and germlings, acts as an invasin through interaction with integrin α_5_β_1_ on host cells and is required for full virulence and lung invasion in corticosteroid-treated mice (16). Beside these virulence determinants on the conidial surface, many allergens are also surface exposed. For example, Asp f 2 has been described as a zinc-acquiring protein and as one of the major allergens of A. fumigatus (17, 18). It was found to bind laminin and IgE antibodies from patients with ABPA (18). Asp f 2 acts as an immune evasion protein by binding human plasma regulators, which leads to inhibition of opsonization and damage of human lung epithelial cells (19). However, most of the surface-exposed proteins are still uncharacterized and a comprehensive picture of the A. fumigatus surface proteome is lacking. Additional studies are necessary to gain a better understanding of the interaction of A. fumigatus conidia with the host.

Several proteomic studies have already been performed to define the proteins associated with the cell surface of A. fumigatus. These studies relied on disruptive extraction methods using strong acids such as formic acid or hydrofluoric acid (HF), alkaline treatment, extraction with SDS buffer at high temperature, or enzymatic treatments with 1,3-β-glucanase, lyticase, chitinase, or trypsin to release surface proteins for detection (9–11, 20–22). Around 300 common proteins were detected in formic acid extracts of dormant A. fumigatus conidia, while the number significantly increased in A. fumigatus mutants lacking either the conidial rodlet layer or the cell wall polymer, α-1,3-glucan (9). The combined approach of HF-pyridine extraction and trypsin shaving revealed 477 different proteins on dormant or swollen conidia (10), while 178 different proteins were detected on the surface of A. fumigatus conidia during germination using the trypsin-shaving approach (11). The many cultivation methods and protein/peptide extraction methods used in the previous studies led to the identification of variable surface proteomes for this fungus (9–11, 20–23).

All the aforementioned methods efficiently extract surface proteins and/or peptides, but they also come with drawbacks; they partially disrupt the surface layer and potentially release cell wall-embedded and cytosolic proteins and peptides in addition to their target cell surface proteins. Cell surface biotinylation has been shown to yield a low rate of contamination by cytoplasmic proteins (24) and in particular provides useful information about the surface-exposed protein regions.

Here, we used a widely applied amine-reactive biotinylation method for surface proteomics (16, 25, 26) to label the exposed lysine or N-terminal amino acid residues (AAs) of A. fumigatus cell surface proteins. Subsequently, liquid chromatography-tandem mass spectrometry (LC-MS/MS) analysis was applied to detect streptavidin-enriched proteins and to define surface-exposed regions based on lysine modifications. The surface localization of several detected proteins was verified through immunofluorescence of Myc-tagged transformants of A. fumigatus. Our report complements the picture of the A. fumigatus surface proteome and reveals surface proteins that may serve as detection markers or targets for immunotherapy in the future. Most importantly, we may have identified a number of proteins that are likely directly involved in the interaction of A. fumigatus and the human host.

## RESULTS

### Biotinylation of A. fumigatus surface proteins.

**FIG 1:**
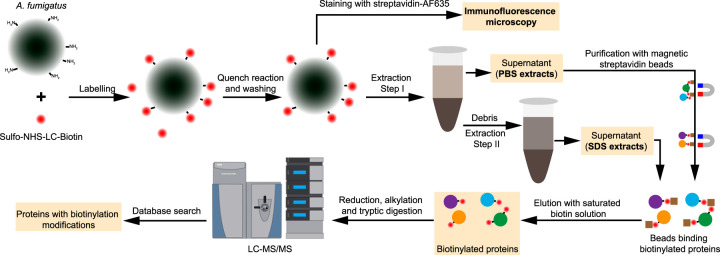
Flowchart for the biotinylation and purification of A. fumigatus surface proteins. The procedure started with covalent labeling of surface proteins with Sulfo-NHS-LC-Biotin for 30 min at 4°C. The fungal material was broken with glass beads in PBS buffer to release the loosely attached surface proteins (PBS extracts). Some noncovalently linked cell wall proteins were extracted using SDS buffer. Biotinylated proteins were purified using magnetic streptavidin beads and then analyzed by LC-MS/MS.

**FIG 2:**
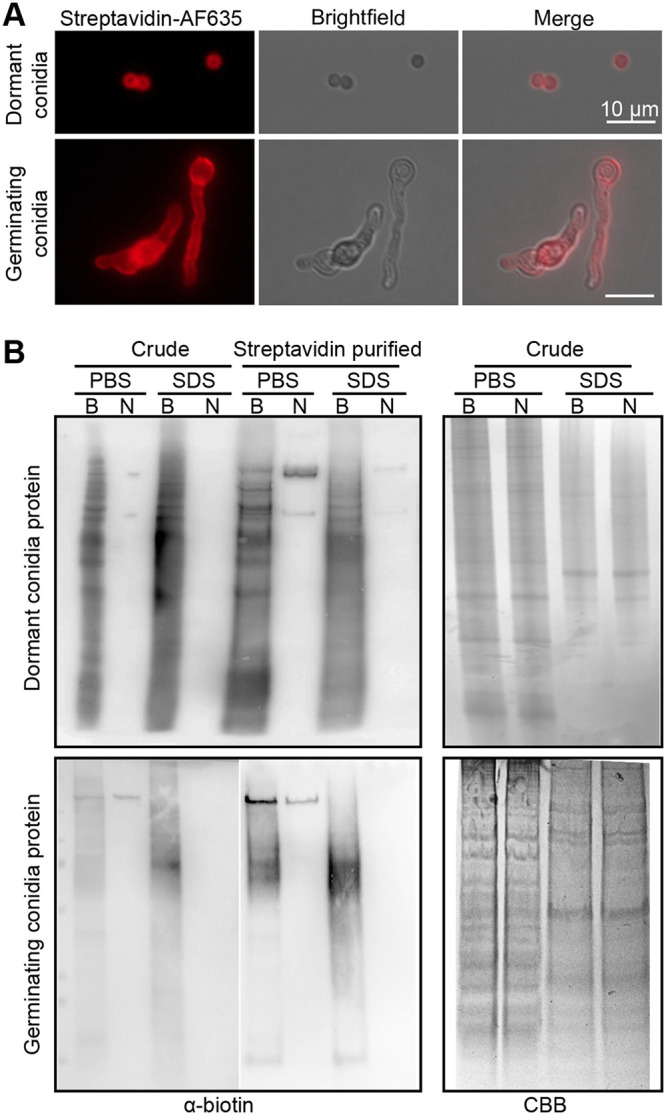
Detection of A. fumigatus surface protein biotinylation. (A) Immunofluorescence staining of A. fumigatus dormant conidia and germinating conidia with Alexa Fluor 635-conjugated streptavidin (Streptavidin-AF635). Scale bar is 10 μm. (B) Immunoblotting analysis of the crude protein extracts and purified proteins from dormant and germinating conidia. B, biotinylated; N, nonbiotinylated; PBS, PBS extracts; SDS, SDS buffer extracts; CBB, Coomassie brilliant blue.

In this study, we sought to expand the repertoire of A. fumigatus surface proteins and their surface-exposed epitopes, which could directly mediate pathogen-host interaction and serve as targets for diagnosis and therapy. We used the biotinylation reagent sulfosuccinimidyl-6-(biotinamido)hexanoate (Sulfo-NHS-LC-Biotin) to label the surface-exposed primary amine residues (e.g., the ε-amino group of lysine residues) throughout germination of A. fumigatus conidia (Fig. 1). The water-soluble and negatively charged reagent Sulfo-NHS-LC-Biotin is one of the most frequently applied biotinylation reagents, particularly in regard to surface proteomics (25–27). Samples were collected from dormant conidia grown at 37°C on *Aspergillus* minimal medium (AMM) agar plates, including swollen conidia (5 h), germlings (8 h), and hyphae (14 h) germinated at 37°C in RPMI liquid medium, as described previously (11). Although there is a possibility of biotin reagent permeation into the fungal cells (26), the biotinylation signal was confined mainly to the surface of conidia and hyphae as observed by immunofluorescence staining against biotinylated proteins (Fig. 2A; see also Fig. S1 in the supplemental material). Due to the high rigidity of the A. fumigatus cell wall, the fungal samples were first disrupted by glass beads in phosphate-buffered saline (PBS) buffer to release loosely attached surface proteins. This was followed by a second step using SDS buffer extraction to remove non-covalently bound hydrophobic surface proteins (Fig. 1). After purification, the samples were checked for the level of protein biotinylation and recovery from the streptavidin beads by Western blotting (Fig. 2B; see also Fig. S2). Proteins that differed widely in molecular mass were detected, demonstrating the ability to label a wide range of proteins using this approach. We also observed biotinylated protein bands in nonbiotinylated control samples (Fig. 2B; see also Fig. S2). Most likely, these represented the three known biotin-dependent carboxylases in A. fumigatus, encoded by Afu4g07710, Afu2g08670, and Afu5g08910, each of which showed high abundance according to the observed peptide spectrum matches (PSM; mass spectral count of repeated peptide identifications that serve as an approximate measure of the relative protein abundance) in our LC-MS/MS analyses (see Data Set S1 in the supplemental material). They are easily distinguishable from the NHS-LC-Biotin modification, which adds 339 Da of mass to the modified proteins.

10.1128/mSphere.00535-20.1 FIG S1 Immunofluorescence staining of biotinylated (A) and nonbiotinylated (B) A. fumigatus morphotypes with Alexa Fluor 635-conjugated streptavidin (Streptavidin-AF635). Germlings indicated with dashed boxes are also shown in Fig. 2A. Scale bar is 20 μm. Download FIG S1, TIF file, 1.5 MB. Copyright © 2020 Jia et al. 2020 Jia et al. This content is distributed under the terms of the Creative Commons Attribution 4.0 International license.

10.1128/mSphere.00535-20.2 FIG S2 Immunoblotting analysis of swollen conidia and mycelia protein extracts. Crude protein extracts (crude) and purified proteins (streptavidin purified) from PBS buffer (PBS) and SDS buffer (SDS) were separated on SDS-PAGE and then transferred to PVDF membranes. Biotinylated proteins were detected with Pierce streptavidin-HRP. B, biotinylated; N, nonbiotinylated. Download FIG S2, TIF file, 0.5 MB. Copyright © 2020 Jia et al. 2020 Jia et al. This content is distributed under the terms of the Creative Commons Attribution 4.0 International license.

10.1128/mSphere.00535-20.9 DATA SET S1 Streptavidin-enriched A. fumigatus proteins detected by LC-MS/MS. In the headers, B1, B2, B3, and B4 represent different repeats of biotinylated samples and C1, C2, C3, and C4 represent different repeats of control samples. D, dormant/resting conidia; S, swollen conidia; G, germinating conidia/germlings; M, mycelium. Download Data Set S1, XLSX file, 15.5 MB. Copyright © 2020 Jia et al. 2020 Jia et al. This content is distributed under the terms of the Creative Commons Attribution 4.0 International license.

### Identification of constitutively, and stage-specifically, exposed proteins.

Over the course of germination, 18 to 463 proteins were detected with at least 2 different peptides and/or a PSM value of ≥10 (Fig. S3A). More proteins were identified in the Sulfo-NHS-LC-Biotin-labeled sample group than in the control group, which was treated the same as the biotinylated protein sample group, except for the biotinylation step (Fig. S3A). Unexpectedly, there were still hundreds of proteins detected in the nonbiotinylated controls (e.g., 273 proteins detected from the nonbiotinylated swollen conidia PBS extract). The presence of a significant number of proteins in the control samples may be explained by unspecific binding of proteins to streptavidin, which was also reported in a previous study (28). Nevertheless, compared to the hundreds of biotinylation sites detected in Sulfo-NHS-LC-Biotin-labeled samples, very few sites were detected in the nonbiotinylated samples (Fig. S3B).

10.1128/mSphere.00535-20.3 FIG S3 Number of proteins (A) and biotinylation sites (B) identified in each sample after surface labeling with Sulfo-NHS-LC-biotin (biotinylated) or without labeling (nonbiotinylated control). Biotinylated proteins were enriched by incubation with streptavidin beads. Streptavidin-binding proteins were analyzed by LC-MS/MS. See also Data Set 1. Download FIG S3, EPS file, 0.7 MB. Copyright © 2020 Jia et al. 2020 Jia et al. This content is distributed under the terms of the Creative Commons Attribution 4.0 International license.

**FIG 3:**
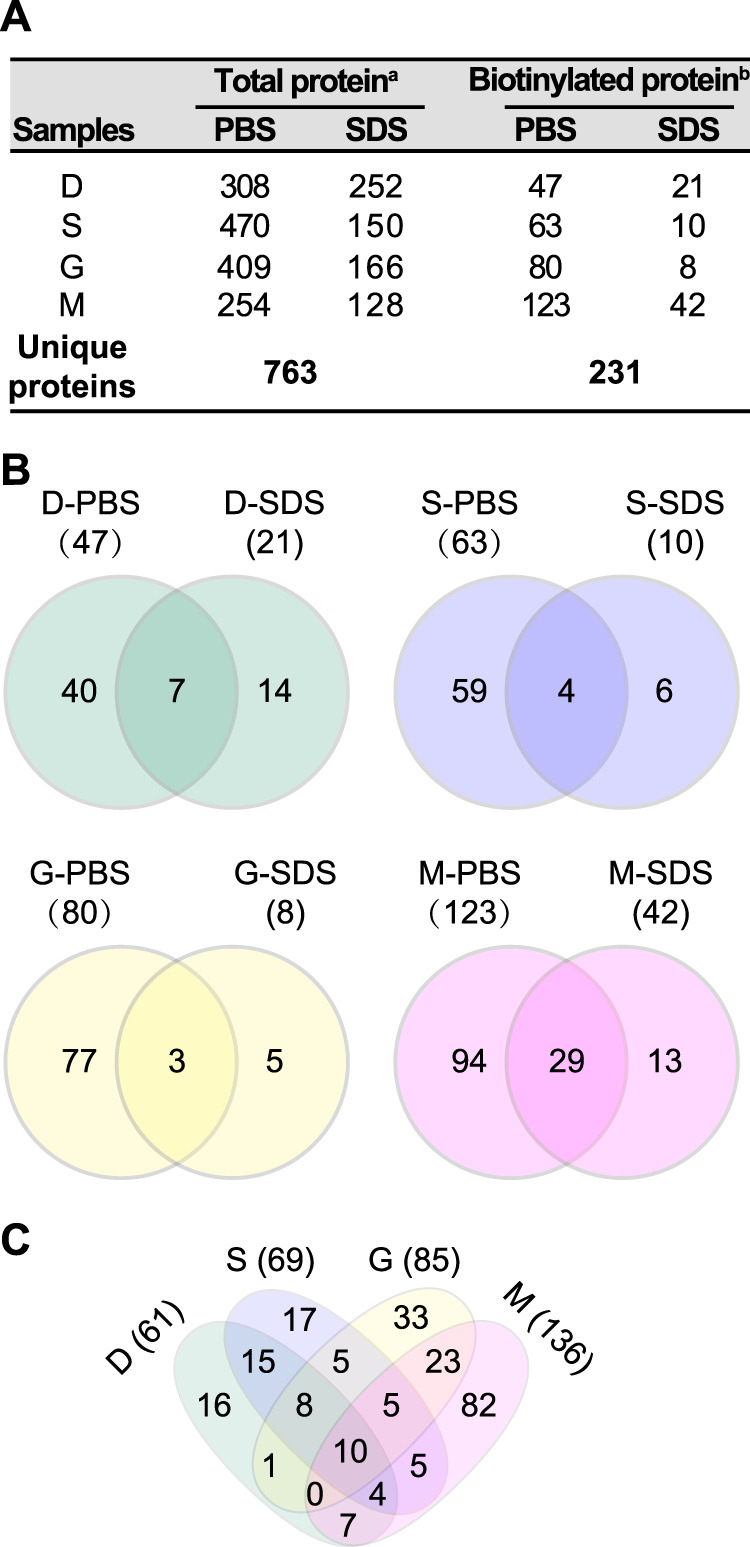
Overview of the surface proteome identified by biotinylation coupled with LC-MS/MS analysis. (A) Number of proteins identified in different samples. Data labeled with a superscript “a” represent numbers of proteins identified by at least two peptides or with numbers of PSMs (peptide spectrum matches) of ≥10. Data labeled with a superscript “b” represent numbers of proteins detected with at least one peptide with biotinylation modification. (B and C) Venn diagrams showing the common and specific surface proteins with biotinylation across different extractions (B) and developmental stages (C).

LC-MS/MS analysis identified in total 763 different A. fumigatus proteins with at least 2 detected peptides and/or a PSM value of ≥10 at the different developmental stages independently of biotinylation treatment (Fig. 3A). To exclude false-positive hits due to unspecific binding, we considered only biotinylated proteins in our analyses. Considering all germination conditions, approximately 30% (231) of the detected proteins had a detectable NHS-LC-Biotin modification (Fig. 3A). Note that many of these proteins were also detected in the nonbiotinylated control samples without biotinylation modifications (see Data Set S1). Fifty-nine (26%) biotinylated proteins had a predicted signal peptide, and 16 (7%) proteins had at least one transmembrane domain (see Table S1 in the supplemental material) (https://fungidb.org/fungidb/ [29]), which demonstrates the enrichment of extracellular proteins in the biotinylated fraction.

10.1128/mSphere.00535-20.6 TABLE S1 Core surfome of A. fumigatus. Download Table S1, XLSX file, 0.2 MB. Copyright © 2020 Jia et al. 2020 Jia et al. This content is distributed under the terms of the Creative Commons Attribution 4.0 International license.

Over the course of germination, 61 proteins with biotinylation were identified on dormant conidia, 69 on swollen conidia, 85 on germlings, and 136 on hyphae. Most of the proteins detected with biotin modifications were found in PBS extracts; only a few (5 to 14) were exclusively found in SDS extracts (Fig. 3A and B; see also Data Set S1). In accordance with our previous surface proteomics study based on a trypsin-shaving approach (11), our data confirm the dynamic change of the surface-exposed proteome of A. fumigatus across germination. There were 16, 17, 33, and 82 proteins detected exclusively on dormant conidia, swollen conidia, germinating conidia, and hyphae, respectively (Fig. 3C). The very different surfome compositions that we observed compared to our previous study (11) can potentially be explained in several ways. First, trypsin is more prone to disturbing the fungal surface structure and thereby releasing peptides of proteins embedded in the cell wall; second, some surface proteins may not be accessible to enzymatic cleavage or biotinylation reactions; third, proteins are inefficiently released from streptavidin beads.

Throughout germination, 10 proteins were biotinylated in all four stages (Table 1), including histone H2B Htb1, histone H2A ortholog Afu3g05360, putative ATP synthase F1 beta subunit Afu5g10550, putative dihydrolipoamide dehydrogenase Afu2g02100, putative aspartate aminotransferase Afu4g10410, and putative triosephosphate isomerase Afu5g13450 and several previously described surface proteins, including translational elongation factor 1 alpha Tef1 (30), peptidase DppV (31), 1,3-beta-glucanosyltransferase Bgt2 (32), and 14-3-3 protein ArtA (30). Biotinylation of Tef1 was detected at sites K472, K476, K483, and K486, indicating that the C terminus of Tef1 was exposed on the conidial and mycelial surface. Except ArtA, all the other proteins had at least two biotinylation sites detected (Table 1). Biotinylation of DppV was detected at sites K136, K239, K327, K328, K351, K373, K423, and K702. Biotinylation of Bgt2 was detected at sites K139, K225, and K231 (Table 1).

**TABLE 1:** Proteins identified in all stages throughout germination in the streptavidin-enriched fractions

Protein name	Brief description	No. of AAs	Biotinylation site(s)	Detected in the samples^*a*^			
Tef1	Putative translation elongation factor EF-1 alpha subunit	494	K472	D	S	—	M
			K476	D	S	G	—
			K483	D	S	G	M
			K486	—	—	G	—
							
Htb1	Histone H2B	140	K9	—	—	G	M
			K20	D	—	G	M
			K25	D	—	G	—
			K31; K134	D	S	G	M
			K60	—	—	—	M
			K99; K130	D	—	—	—
			K100	D	—	—	M
							
Afu5g10550	ATP synthase F1, beta subunit	519	K122	D	S	G	M
			K148	—	S	G	—
			K151	—	S	—	—
			K338; K392; K473	—	—	G	—
							
DppV	Secreted dipeptidyl-peptidase	721	K136; K351; K373; K423	—	—	—	M
			K239; K328	—	—	G	M
			K327	—	S	G	M
			K702	D	—	G	—
							
Bgt2	Cell wall glucanase	446	K139; K231	—	—	—	M
			K225	D	S	G	M
							
ArtA	14-3-3 family protein	261	N-term	D	S	G	M
							
Afu2g02100	Putative dihydrolipoamide dehydrogenase	513	K204; K269; K383	—	—	G	—
			K273; K283; K302; K351	—	—	—	M
			K286; K420	—	—	G	M
			K435	D	S	G	M
							
Afu3g05360	Has domain(s) with predicted DNA binding, protein heterodimerization activity	265	K9; K76; K77	—	—	—	M
			K14	D	—	G	M
			K22	D	S	—	M
			K38	D	—	—	M
							
Afu4g10410	Putative aspartate aminotransferase	429	K72	—	—	G	M
			K85	D	S	—	—
							
Afu5g13450	Putative triosephosphate isomerase	249	K102	—	S	—	—
			K137	D	—	—	—
			K216	—	—	G	M

a Lysine residues of the protein detected with biotinylation in dormant (D), swollen (S), or germinating (G) conidia or hyphae (M) and not detected (—) are indicated.

Htb1, Tef1, and Afu5g10550 were also abundantly detected throughout germination (Table 2). Somewhat surprisingly, the histone H2B (Htb1, Afu3g05350) seemed to be one of the most abundant proteins throughout the germination time course (Table 1 and 2). The number of PSM/length of Htb1 in dormant conidia (3.25) was even higher than that determined for RodA (2.37) (Table 2), in contrast to previous studies performed using different approaches (10, 11, 21). One possible explanation for this discrepancy is the difference in the total number of the modifiable lysines in each protein. There are 23 lysine residues in the 140 amino acid residues (AAs) of Htb1, 9 of which were detected with a biotinylation modification (Table 1). In contrast, there are only 9 lysine residues present in the hydrophobic protein RodA. RodA has been shown to be present throughout germination by previous studies (11, 21); this could be explained by contamination with ungerminated conidia or by a low level of RodA in hyphae. In this study, RodA was abundantly detected in dormant, swollen, and germinating conidia (Table 2). Three sites, including K50, K55, and K126, were detected with biotinylation marks. All three lysine residues are localized in the relatively hydrophilic α-helical regions of the protein (33), suggesting that only the surface-exposed lysine residues are likely to be biotinylated.

**TABLE 2:** LC-MS/MS analysis of highly abundant proteins detected throughout the germination course (top 15 of each morphotype)^*a*^

Protein	Protein description	No. of AAs	No. of PSMs/length of protein				Biotinylation site(s)^*b*^
			D	S	G	M	
Htb1^*c*^	Histone H2B	140	3.25	0.96	1.35	1.23	See Table 1
Afu5g10550^*c*^	ATP synthase F1, beta subunit	519	0.91	2.44	1.49		See Table 1
RodA^*c*^	Conidial hydrophobin	159	2.37	3.70	2.75		K50^D,^^S,^^G^; K55^D,^^S^; K126^D,^^S^
GpdA^*c*^	Glyceraldehyde-3-phosphate dehydrogenase	338	2.88	3.58			K194^D,^^M^; K215^D,^^S,^^M^
Tef1^*c*^	Translation elongation factor EF-1 alpha subunit	494	1.60	2.79			See Table 1
UbiA*	Ubiquitin	128	1.45	2.32			K6^S^; K33^D,^^S^; K48^D,^^S^
UbiC*	Ubiquitin (Afu3g11260), putative	154	1.49	3.17			K6^S^; K11^S^; K33^D,^^S^; K48^D,^^S^
Pil1	Cell wall integrity signaling protein	345	1.09	1.08			K29^D^; K45^D,^^S^; K131^D,^^S^; K160^D,^^S^; K270^D^
Sod1^*c*^	Cu/Zn superoxide dismutase	154	0.86	1.86			K43^D,^^S,^^G^
Afu7g01060	Cysteine-rich secreted protein	343	0.71	1.01			K212^D,^^S^; K224^D,^^S^
Grg1^*c*^	Glucose-repressible gene	69	1.22				K28 or K32^S^; K32^D^; K46^D,^^S^
ConJ^*c*^	Protein of unknown function	83	1.15				N-Term^D^; K48^D^
Afu2g13860	Histone H4	142	0.92				K71^D,^^M^; K117^M^
Scf1^*c*^	Putative heat shock protein	89	0.81				K81^D^
Ecm33	GPI-anchored cell wall organization protein	398	0.67				K170^S,^^M^; K306^D^; K334^M^
CatA	Spore-specific catalase	750	0.63				K534 or K537^D,^^S^; K608 or K612^S^
Afu8g05320^*c*^	Putative mitochondrial F1 ATPase subunit alpha	556		3.87	2.63		K135^S^; K164^G^; K170^G,^^M^; K170 or K172^S^; K233^G^; K243^G^; K427^G,^^M^; K430^G^; K531^G^
Asp f MDH^*c*^	Putative NAD-dependent malate dehydrogenase	340		1.27	2.10		K91^G^; K185 or K186^M^; K238^S,^^G,^^M^; K263^S,^^G,^^M^; K269^G^; K303^G^; K309^G,^^M^; K327^M^; K331^M^
CpcB	G-protein complex beta subunit	316		0.99	1.31		K38^G^; K56^D,^^S,^^G^; K172^G^; K277^S,^^G^
Bgt1	Putative 1,3-beta-glucanosyltransferase	305		1.98			K36^G^; K51^S,^^M^; K69^S^; K125^S,^^G,^^M^; K141^S,^^G,^^M^; K145^S^; K275^S^; K304^S,^^G,^^M^
Afu1g04070	Eukaryotic initiation factor 5A	157		1.08			K40^S^; K69^S^
RodB	Conidial cell wall hydrophobin	183		0.90			K105^S^
Afu4g07710	Putative pyruvate carboxylase	1193			8.38	1.61	K571^M^; K684^M^; K1004^M^; K1157^G^
Afu6g13250^*c*^	60S ribosomal protein L31e	123			3.55	1.98	K30^M^; K59^M^; K69^M^; K70^G^; K111^G^
Afu3g00880	Putative adhesin protein	219			2.61	1.18	K24^S,^^G,^^M^; K63^S,^^G,^^M^
Afu4g04460	60S ribosomal protein L13	226			6.07		K225^G^; K226^D^
Afu3g12300	60S ribosomal protein L22	124			2.39		K17^G^
Hsp70^*c*^	Heat shock protein	638			1.75		K54^M^; K185^G^; K244^M^; K249^G,^^M^; K421^G^; K449^M^; K498^G,^^M^; K505^G,^^M^; K510^G,^^M^; K522^G^; K555^M^
PgkA^*c*^	Putative phosphoglycerate kinase	417			1.35		K32^G^; K33^G^; K79^G^; K246^G^; K264^G^
Sod3/Asp f 6	Manganese superoxide dismutase	210			1.28		K50^G^; K59^G^; K88^G,^^M^; K93^G,^^M^; K101^G^; K202^M^
Ndk1	Putative nucleoside diphosphate kinase	153			1.23		K84^G,^^M^
Afu4g07730	60S ribosomal protein L11	176				3.11	K79^M^; K91^M^; K157^M^
Afu3g06960	60S ribosomal protein L21	158				2.18	K20^M^; K107^M^; K110^M^
Gel2/Asp f GT	GPI-anchored 1,3-beta-glucanosyltransferase	475				1.91	K36^M^; K187^M^; K372^M^; K379^S,^^M^; K388^M^; K393^M^
Afu1g05390	ATP:ADP antiporter activity	308				1.48	K103^M^; K146^G,^^M^; K149^M^; K252^M^; K264^M^
Afu2g09200	60S ribosomal protein L30	106				1.32	K34^M^
Asp f 4	Allergen Asp f 4	322				1.29	K140^S,^^M^; K155 or 156^M^; K220^M^; K306^M^
BtgE	Putative cell wall glucanase	616				1.25	K376^M^; K387^M^; K389^M^; K577^G,^^M^
Afu3g06840	Cytosolic small ribosomal subunit S4	261				1.23	K106^M^
Afu1g11130	60S ribosomal protein L6	200				1.21	K107^M^
GliT	Gliotoxin sulfhydryl oxidase	334				1.19	K117^M^; K227^M^
Afu6g12990^*c*^	Cytosolic large ribosomal subunit protein L7A	263				1.16	K8^M^; K212^M^; K229^M^; K255^S^

a Values listed represent the top 15 proteins with the highest PSM/length values for each developmental stage. For stages D and S, 16 proteins are listed, including UbiA and UbiC (indicated by an asterisk [*]), which have multiple shared peptides.

b Lysine residues of the protein detected with biotinylation marks in dormant (D), swollen (S), or germinating (G) conidia or hyphae (M) are indicated.

c Proteins commonly detected as demonstrated by different surface proteomics data; see also Table 5.

### Immunoreactive proteins are exposed on the surface of A. fumigatus.

In a unique subset of individuals with atopic allergy, sensitization to A. fumigatus allergens can develop into allergic asthma, allergic sinusitis, and, following fungal lung colonization, into ABPA (1). Twenty-three different allergens have been reported in accordance with the systematic allergen nomenclature (www.allergome.org), but actually more than 100 immunoreactive A. fumigatus proteins have already been uncovered by immunoproteomic studies (5). In our work, 20 allergens (Table 3) were detected by surface biotinylation. Allergens Asp f 17, Asp f 18, Asp f 27, Asp f mannosidase, Asp f catalase, and Asp f glucosidase were found on dormant conidia. Biotinylation of Asp f 27 K140 was detected on dormant, swollen, and germinating conidia. In addition to Asp f 27, Asp f MDH was identified on swollen conidia, germlings, and hyphae. Biotinylation of Asp f MDH K238 and of K263 was detected on the three different morphologies of A. fumigatus. Nine allergens were found only on germlings and/or hyphae (Table 3). Consistent with the literature, these data again clearly demonstrate that numerous known A. fumigatus allergens are exposed on the fungal surface (9–11, 19, 34).

**TABLE 3:** A. fumigatus allergens exposed to the surface

Allergen^*a*^	Protein description	No. of AAs	Detected in the samples	Biotinylation site(s)^*b*^
Asp f 1	Mitogillin	176	G	K96^G^; K138^G^; K140^G^
Asp f 2	Allergen Asp f 2	314	M	K83^M^
Asp f 4	Allergen Asp f 4	322	S, M	K140^S,^^M^; K155 or 156^M^; K220^M^; K306^M^
Asp f 6 (Sod3)	Putative manganese superoxide dismutase	210	G, M	K50^G^; K59^G^; K88^G,^^M^; K93^G,^^M^; K101^G^; K202^M^
Asp f 9	Cell wall glucanase	395	S, M	K190^S,^^M^
Asp f 11 (Cyp4)	Putative cyclophilin	205	S	K119^S^
Asp f 12 (Hsp90)	Heat shock protein	706	G	K481^G^
Asp f 17 (Mp1)^*c*^	Putative cell wall galactomannoprotein	284	D	K63^D^; K144^D^
Asp f 18 (Alp2)	Autophagic (vacuolar) serine protease	495	D	K261^D^; K268^D^; K271^D^; K291^D^
Asp f 23 (RpL3)	Allergenic ribosomal L3 protein	392	M	K5^M^
Asp f 27^*c*^	Putative peptidyl-prolyl *cis*-*trans* isomerase; cyclophilin	163	D, S, G	K43^D,^^S^; K140^D,^^S,^^G^; K152 or K153^D^
Asp f 28^*c*^	Putative thioredoxin	171	M	K125^M^
Asp f mannosidase (MsdS)^*c*^	Putative 1,2-alpha-mannosidase	503	D	K212^D^; K274^D^; K415^D^; K416^D^; K488^D^
Asp f catalase (Cat1)^*c*^	Catalase	728	D	K261^D^; K346^D^; K466^D^
Asp f glucosidase (Exg12)^*c*^	Secreted beta-glucosidase	863	D, M	K127^D^; K178^D^; K415^D^; K603^D^; K839^D^; K843^D,^^M^
Asp f FDH	Putative NAD-dependent formate dehydrogenase	418	G, M	K304^M^; K319^G^; K327^M^
Asp f MDH^*c*^	Putative NAD-dependent malate dehydrogenase	340	S, G, M	K91^G^; K185 or K186^M^; K238^S,^^G,^^M^; K263^S,^^G,^^M^; K269^G^; K303^G^; K309^G,^^M^; K327^M^; K331^M^
Asp f GT (Gel2)	GPI-anchored 1,3-beta-glucanosyltransferase	475	S, M	K36^M^; K187^M^; K372^M^; K379^S,^^M^; K388^M^; K393^M^
Asp f gamma_Actin (Act1)^*c*^	Actin	393	M	K346^M^
Asp f RPS3^*c*^	40S ribosomal protein S3	266	M	K78^M^

a A. fumigatus allergens based on allergome website (www.allergome.org).

b Lysine residues of the protein detected with biotinylation marks in dormant (D), swollen (S), or germinating (G) conidia and hyphae (M) are indicated.

c Allergens commonly detected in different surface proteomics data; see also Table 5.

In addition to the allergens, there were also other immunoreactive proteins that could serve as biomarkers for diagnosis or targets for immunotherapy (35–37). Our analysis revealed biotinylation of DppV, Bgt2, and Afu5g10550 in all four morphotypes (Table 1). Afu5g10550 encodes an ATP synthase F1 beta subunit, which reacts with immunosera from rabbits exposed to A. fumigatus conidia (35). Biotinylation of Afu5g10550 K122 was detected throughout germination. It was also one of the most prevalent proteins found on dormant, swollen, and germinating conidia (Table 1 and 2). Immunoreactive GpdA, Asp f MDH, Bgt1, Asp f 6, Hsp70, PgkA, Asp f 4, GliT, and Asp f GT proteins were also prevalent on the surface of one or two morphological stages (Table 2).

### Heat shock proteins are exposed to the surface.

Heat shock proteins (HSPs) and a large set of cochaperones are ubiquitous molecular chaperones that act in maintaining protein homeostasis (38). Although they represent highly abundant housekeeping proteins, it has been well established that HSPs are also secreted extracellularly or localized on the cell surface (39), where they exhibit moonlighting function. Hsp90, also known as Asp f 12, is detectable on the cell wall of A. fumigatus (34) and was also found to be biotinylated in this study (Table 3). Considering the detection of biotinylated Hsp90 and Hsp70, we investigated whether other chaperone-related proteins are present on the cell surface of A. fumigatus. Indeed, at least 14 chaperone-related proteins were detected with biotinylation modifications (Table 4). Most of the chaperones were detected on the surface of germlings or hyphae with the exception of Scf1 and GrpE. Scf1 shows similarities to the 12-kDa heat shock protein of Saccharomyces cerevisiae and was biotinylated at K81 (Table 4). It was found to be one of the most prevalent proteins on dormant conidia of A. fumigatus strains CEA10 (Table 2) and ATCC 46645 (10). Five chaperones (Hsp70, Hsp88, HscA, BipA, and Ssc70) belonging to the Hsp70 family were detected in our study (Table 4). In addition to Hsp70, four other HSPs (Sti1, Hsp88, HscA, and Ssc70) have been shown to induce serological antibody responses in ABPA patients (37).

**TABLE 4:** Chaperones and cochaperones with cell surface localization

Protein	Accession no.	Protein description	No. of AAs	Detected in the samples	Biotinylation site(s)^*a*^
Scf1	Afu1g17370	Putative heat shock protein	89	D	K81
GrpE	Afu2g13040	Mitochondrial cochaperone	250	D, S	K99^D,S^
Wos2	Afu5g13920	Putative Hsp90 binding cochaperone	201	S, G	K35^G^; K193^S^
Hsp60	Afu2g09290	Putative antigenic mitochondrial protein	587	G	K510
Hsp90/Asp f 12	Afu5g04170	Heat shock protein	706	G	K481
Afu6g10700	Afu6g10700	Ortholog(s) have chaperone binding, unfolded protein binding activity	122	G	K50; K56
Sti1	Afu7g01860	Putative heat shock protein	581	G	K232
Hsp70	Afu1g07440	Molecular chaperone	638	G, M	See Table 2
Hsp88	Afu1g12610	Hsp70 chaperone	714	G, M	K130 or K133^M^; K216^M^; K260^M^; K271^G,M^
HscA	Afu8g03930	Putative Hsp70 chaperone	614	G, M	K59^M^; K429^G^; K498^G^; K531^G^; K539^G^; K566 or K568^G^; K609^G^
BipA	Afu2g04620	Hsp70 chaperone	672	G, M	K133^M^; K549^G,M^
Ssc70	Afu2g09960	Putative mitochondrial Hsp70 chaperone	661	G, M	K92^G,M^; K113^m^; K124^G,M^; K192^G^; K277^M^; K278^M^; K453^G^
ClxA	Afu4g12850	Calnexin, endoplasmic reticulum (ER) membrane chaperone	563	M	K153
Egd2	Afu6g03820	Nascent polypeptide-associated complex subunit alpha	204	M	K50; K51; K62

a Lysine residues of the protein detected with biotinylation marks in dormant (D), swollen (S), or germinating (G) conidia or hyphae (M) are indicated.

**FIG 4:**
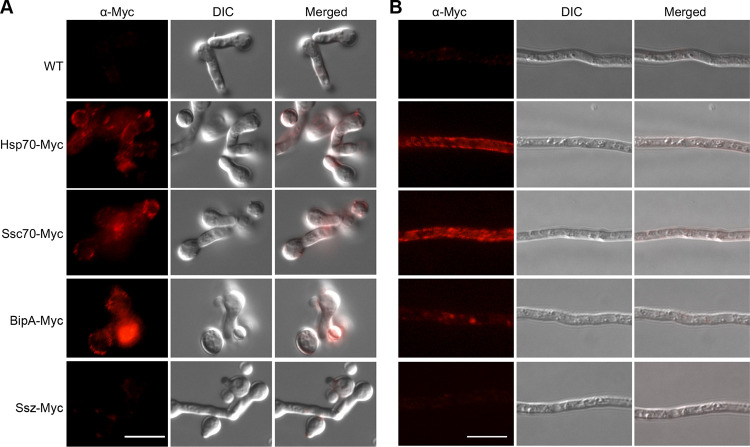
Immunofluorescence staining of A. fumigatus. Germlings (A) and hyphae (B) were blocked in PBS–1% (wt/vol) BSA and then incubated with the primary anti-Myc antibody. The surface Myc-tagged Hsp70s were indirectly detected with an AF568-conjugated secondary antibody. Scale bar = 10 μm. WT, wild type.

To confirm the surface localization of the HSPs, we generated constructs by fusing a Myc tag to the C terminus of the HSPs. These constructs were ectopically integrated into the genome of A. fumigatus (Table S2 and S3). The *hsp70-Myc*, *ssc70-Myc*, *bipA-Myc*, and *ssz-Myc* transformants were verified by immunoblotting using an anti-Myc tag antibody (Fig. S4). Using immunofluorescence microscopy, we found that all of the Myc-tagged fusion proteins were localized on the surface of germinating conidia (Fig. 4A) and hyphae (Fig. 4B), except for the negative control, the HSP70 chaperone Ssz (Afu2g02320), which was not detected on the surface in our biotinylation experiment. This independently confirms that three HSP70 chaperones of A. fumigatus were able to be localized to the cell surface of the fungus.

10.1128/mSphere.00535-20.4 FIG S4 Immunoblotting of A. fumigatus protein extracts. Myc-tagged proteins were detected using anti-Myc antibody. For the A. fumigatus strains expressing *hsp70-Myc* and *ssc70-Myc*, protein was extracted from dormant conidia; for the strains expressing *ssz-Myc* and *bipA-Myc*, protein was extracted from mycelium. An anti-GAPDH antibody served as a loading control. Download FIG S4, TIF file, 0.4 MB. Copyright © 2020 Jia et al. 2020 Jia et al. This content is distributed under the terms of the Creative Commons Attribution 4.0 International license.

10.1128/mSphere.00535-20.7 TABLE S2 A. fumigatus strains used in this study. Download Table S2, DOCX file, 0.01 MB. Copyright © 2020 Jia et al. 2020 Jia et al. This content is distributed under the terms of the Creative Commons Attribution 4.0 International license.

10.1128/mSphere.00535-20.8 TABLE S3 Oligonucleotides used in this study. Download Table S3, DOCX file, 0.01 MB. Copyright © 2020 Jia et al. 2020 Jia et al. This content is distributed under the terms of the Creative Commons Attribution 4.0 International license.

### The core surface proteome (surfome) of A. fumigatus.

**FIG 5:**
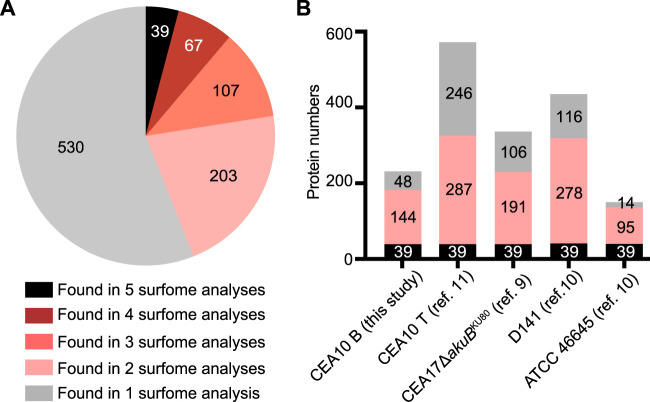
The number of A. fumigatus surface proteins identified in different surface proteomics experiments. (A) Pie chart showing the number of proteins present in different experiments. (B) Each of the bars represents the number of identified proteins, with black zones highlighting the number of proteins present in all of the surfome data available (see also Table 5), including the results obtained in this study (CEA10 B, corresponding to all the morphotypes of CEA10 obtained using the biotinylation method); the surfome of CEA10 (CEA10 T, corresponding to all the morphotypes of CEA10 obtained using the trypsin-shaving method); D141 (dormant and swollen conidia) results obtained using the trypsin shaving method (10, 11); the CEA17Δ*akuB*^KU80^ dormant conidia surfome results obtained using the formic acid extract (9); and the ATCC 46645 surfome detected by hydrogen fluoride-pyridine extraction and trypsin shaving (10). The pink zones highlight the number of proteins present in at least two sets of surfome data but not all sets of surfome data. Gray zones indicate the number of proteins present in just one experiment.

Considering that several surface proteomic studies have been performed using different methods (trypsin shaving, HF/pyridine, and formic acid extraction), we attempted to compare these data sets to provide a more comprehensive picture of the A. fumigatus surfome (9–11). In total, 946 different proteins were detected as surface proteins in this study and the aforementioned studies, including 416 proteins that were detected by at least two approaches (Fig. 5; see also Table S1). A total of 39 proteins were commonly identified in all studies (Fig. 5) (Table 5; see also Table S1). These 39 proteins included 15 of the most prevalent proteins identified in our study and 9 allergens (Tables 2, 3, and 5; see also Table S1). Two small proteins, Grg1 (glucose-repressible gene) and ConJ (conidiation-specific protein 10), were detected among the most prevalent proteins on dormant conidia of the ATCC 46645 strain using the HF-pyridine extraction method (10), on the CEA10 strain using the trypsin-shaving method (11), and in this study using biotinylation (Table 2 and 5). Grg1 was detected with biotinylation at the positions K32 and K46, while ConJ was biotinylated at the N terminus and site K48 (Table 2 and 5). Conidial surface protein CcpA, which contributes to fungal virulence, was detected with biotinylations at positions K41 and K90 (Table 5). Putative glycosylphosphatidylinositol (GPI)-anchored cell wall protein CweA (11) was detected with a biotinylation marker at site K358 (Table 5). In addition to the 39 commonly detected proteins, 67 proteins were detected in at least four surfome data sets (Fig. 5A), 39 of which were detected with biotinylated amino groups (Table S1), including five allergens (Asp f 1, Asp f 4, Asp f 11, Asp f 12, and Asp f 18) and seven other prevalent proteins (1,3-beta-glucanosyltransferase Bgt1, cell wall protein Ecm33, nucleoside kinase Ndk1, eukaryotic initiation factor 5A [Afu1g04070], a putative ADP/ATP carrier [Afu1g05390], the 60S ribosomal protein L13 [Afu4g04460], and ubiquitin [Afu3g11260]) (Table 2). In total, 183 surface proteins with biotinylation modifications identified in this study were also detected by alternative methods (Fig. 5B). All in all, the core surfome of A. fumigatus provides a valuable database of protein targets for further studies on host-pathogen interactions, diagnosis, and immunotherapy.

**TABLE 5:** The 39 most commonly identified proteins^*a*^

Protein	Accession no.	Protein description	No. of AAs	Biotinylation site(s)^*b*^	Note^*c*^
Htb1	Afu3g05350	Histone H2B	140	K9^G,^^M^; K20^D,^^G,^^M^; K25^G^; K31^D,^^S,^^G,^^M^; K60^M^; K99^D^; K100^D,^^M^; K130^D^; K134^D,^^S,^^G,^^M^	See Tables 1 and 2
Tef1	Afu1g06390	Putative translation elongation factor EF-1 alpha subunit	494	K472^D,^^S,^^M^; K476^D,^^S,^^G^; K483^D,^^S,^^G,^^M^; K486^G^	See Tables 1 and 2
Afu5g10550	Afu5g10550	ATP synthase F1, beta subunit	519	K122^D,^^S,^^G,^^M^; K148^S,^^G^; K151^S^; K338^G^; K392^G^; K473^G^	See Tables 1 and 2
ArtA	Afu2g03290	14-3-3 family protein	261	N-term	See Table 1
Bgt2	Afu3g00270	Cell wall glucanase	446	K139^M^; K231^M^; K225^D,^^S,^^G,^^M^	See Table 1
Asp f MDH	Afu7g05740	Putative NAD-dependent malate dehydrogenase	340	K91^G^; K185 or K186^M^; K238^S,^^G,^^M^; K263^S,^^G,^^M^; K269^G^; K303^G^; K309^G,^^M^; K327^M^; K331^M^	See Tables 2 and 3
Scf1	Afu1g17370	Putative heat shock protein	89	K81^D^	See Tables 2 and 4
Hsp70	Afu1g07440	Molecular chaperone	638	K54^M^; K185^G^; K244^M^; K249^G,^^M^; K421^G^; K449^M^; K498^G,^^M^; K505^G,^^M^; K510^G,^^M^; K522^G^; K555^M^	See Tables 2 and 4
RodA	Afu5g09580	Conidial hydrophobin	159	K50^D,^^S,^^G^; K55^D,^^S^; K126^D,^^S^	See Table 2
Grg1	Afu5g14210	Glucose-repressible gene	69	K28 or K32^S^; K32^D^; K46^D,^^S^	See Table 2
ConJ	Afu6g03210	Protein of unknown function	83	N-Term^D^; K48^D^	See Table 2
Afu6g13250	Afu6g13250	60S ribosomal protein L31e	123	K30^M^; K59^M^; K69^M^; K70^G^; K111^G^	See Table 2
Sod1	Afu5g09240	Cu/Zn superoxide dismutase	154	K43^D,^^S,^^G^	See Table 2
Afu6g12990	Afu6g12990	Cytosolic large ribosomal subunit protein L7A	263	K8^M^; K212^M^; K229^M^; K255^S^	See Table 2
GpdA	Afu5g01970	Glyceraldehyde-3-phosphate dehydrogenase	338	K194^D,^^M^; K215^D,^^S,^^M^	See Table 2
PgkA	Afu1g10350	Putative phosphoglycerate kinase	417	K32^G^; K33^G^; K79^G^; K246^G^; K264^G^	See Table 2
Afu8g05320	Afu8g05320	Putative mitochondrial F1 ATPase subunit alpha	556	K135^S^; K164^G^; K170^G,^^M^; K170 or K172^S^; K233^G^; K243^G^; K427^G,^^M^; K430^G^; K531^G^	See Table 2
Asp f 27	Afu3g07430	Putative peptidyl-prolyl *cis*-*trans* isomerase; cyclophilin	163	K43^D,^^S^; K140^D,^^S,^^G^; K152 or K153^D^	See Table 3
Asp f 28	Afu6g10300	Putative thioredoxin	171	K125^M^	See Table 3
Asp f RPS3	Afu1g05630	40S ribosomal protein S3	266	K78^M^	See Table 3
Asp f 17 (Mp1)	Afu4g03240	Putative cell wall galactomannoprotein	284	K63^D^; K144^D^	See Table 3
Asp f gamma_actin (Act1)	Afu6g04740	Actin	393	K346^M^	See Table 3
Asp f mannosidase (MsdS)	Afu1g14560	Putative 1,2-alpha-mannosidase	503	K212^D^; K274^D^; K415^D^; K416^D^; K488^D^	See Table 3
Asp f catalase (Cat1)	Afu3g02270	Catalase	728	K261^D^; K346^D^; K466^D^	See Table 3
Asp f glucosidase (Exg12)	Afu1g05770	Secreted beta-glucosidase	863	K127^D^; K178^D^; K415^D^; K603^D^; K839^D^; K843^D,^^M^	See Table 3
Nhp6	Afu3g11610	Nonhistone chromosomal protein	104	K64^G^	
HhtA	Afu1g13790	Histone H3	136	K10^D^; K15^D^; K43^M^; K123^M^	
Afu1g13550	Afu1g13550	Protein of unknown function	143	K116^D^; K140^D^	
Afu1g11190	Afu1g11190	Putative eukaryotic translation elongation factor 1 subunit eEF1-beta	227	N-Term^D^; K129^S^; K188^G^	
CcpA	Afu1g13670	Protein of unknown function	235	K41^S,^^G^; K90^S^	
Afu4g06910	Afu4g06910	Putative outer mitochondrial membrane protein porin	284	K47^M^; K93^M^; K107 or K110^M^; K213^M^	
Afu5g02040	Afu5g02040	Putative extracellular lipase	296	K190^D,^^S^; K208^D,^^S^	
Afu5g03540	Afu5g03540	Ortholog(s) have flavin-linked sulfhydryl oxidase activity	386	K287^M^	
Gel1	Afu2g01170	1,3-Beta-glucanosyltransferase with a role in elongation of 1,3-beta-glucan chains	452	K79^M^; K377^M^	
NagA	Afu8g05020	Putative secreted N-acetylhexosaminidase	600	K117^M^	
Afu2g13530	Afu2g13530	Putative translation elongation factor EF-2 subunit	839	N-Term^G^; K308^M^; K492^S^	
CweA	Afu4g09600	Putative GPI-anchored cell wall protein	848	K358^S^	
Afu1g16250	Afu1g16250	Putative alpha-glucosidase B	881	K30^M^	
ExgO	Afu1g14450	Exo-beta-1,3-glucanase;	958	K71^D^; K76^D^; K665^S^	

a Proteins commonly detected in this study (CEA10 B; all the morphotypes of CEA10 using biotinylation method) in the surfome of CEA10 (CEA10 T; all the morphotypes of CEA10 using trypsin shaving method) and of D141 (dormant and swollen conidia) obtained using trypsin shaving method (10, 11), the CEA17Δ*akuB*^KU80^ dormant conidia surfome obtained by formic acid extraction (9), and the ATCC 46645 surfome detected by hydrogen fluoride-pyridine extraction and trypsin shaving (10).

b Lysine residues of the protein detected with biotinylation marks in dormant (D), swollen (S), or germinating (G) conidia or hyphae (M) are indicated.

c Proteins listed in other tables also.

## DISCUSSION

Proteins in combination with other cell wall components on the surface of A. fumigatus conidia play a key role in protecting the fungus from environmental insults and host defense responses during an infection (10, 12, 16, 40, 41). Several methods and techniques have been used to investigate the surface proteins of this pathogenic fungus, leading to the detection of hundreds of surface proteins, whose presence on the surface changes dynamically during development (9–11, 20, 23). Most methods used for the extraction of surface proteins, such as enzymatic treatment with glucanase or trypsin or acidic extraction with formic acid or hydrogen fluoride-pyridine, have the potential to release intracellular or unexposed cell wall proteins. Cell-impermeable biotinylation reagents, which react with primary amines accessible on the cell surface, have successfully been used for labeling and identification of surface proteins with little contamination (16, 25, 26, 42, 43). To further expand the range of A. fumigatus surface proteomes and to solidify the data representing the common core surfome of A. fumigatus, we used a surface biotinylation approach, which had not been applied to A. fumigatus previously. The aim was to characterize and detail the A. fumigatus surfome with surface-exposed regions across germination and verify the surface localization of selected proteins by an additional method (Fig. 4). Therefore, our work provides a multitude of candidates for further investigation of host-pathogen interaction and possible immunodiagnostic/therapeutic usages.

Proteins on the surface mediate direct contacts between pathogens and hosts. In addition to several known surface proteins, such as RodA, CalA, Asp f 2, and CcpA (10, 12, 16, 19), abundant surface proteins detected in this study, such as Tef1, ArtA, Hsp70, and Hsp90, also have the potential to interact with a range of host proteins (44). Candida albicans Tef1 was shown to be surface localized and to be able to bind human plasminogen, probably through C-terminal lysine residues (45). In this study, Tef1 was detected with biotinylation at the C-terminal K472-to-K486 region throughout germination (Table 1), indicating that Tef1 in A. fumigatus might have a similar function. The 14-3-3 proteins, such as ArtA, also have the ability to bind a multitude of proteins and to play an important role in morphogenesis and sensing of environmental cues in fungi (46–48). The presence of HSPs on the surface of mammalian cells and microorganisms has been well documented (39, 49–53) and was also confirmed in this study (Fig. 4). The surface-localized HSPs influence the interactions between viral, bacterial, and fungal pathogens and with host cells as well (49, 54–56). For example, expression of Listeria monocytogenes heat shock protein ClpC, a member of the 100-kDa heat shock protein family, is required for cell adhesion and invasion (53) and also allows this bacterium to escape from the phagosome (52). C. albicans Hsp70 protein Ssa1 is an invasin that binds to host cell cadherins to induce host cell endocytosis, which is critical for C. albicans to cause maximal host cell damage (51). In line with this, A. fumigatus Hsp70 and Hsp90 were predicted to interact with host proteins in conidia containing mouse macrophage phagolysosomes (44), suggesting the potential roles of surface HSPs in manipulating the host immune responses.

In addition to their roles in pathogenicity, surface proteins could also be targets of immunotherapies based on the use of antibodies. Surface proteins are particularly suitable for such use due to their accessibility. The use of antibody-based therapies is rapidly growing, since they represent a promising approach for directly attacking the pathogen and boosting the innate immune system. However, only a few monoclonal antibodies against fungal pathogens have been developed and have advanced to clinical trials (57). One example is Mycograb, an Hsp90-specific antibody fragment, which showed promise for treating *Candida* infections in combination with amphotericin B (58) but failed to obtain marketing authorization. In mouse experiments, treatment with anti-Hsp60 antibodies reduced fungal burden after infection with the dimorphic fungi Histoplasma capsulatum and Paracoccidioides lutzii (59, 60). On the negative side, the amino acid sequences of HSPs are highly conserved and cross-reactivity can occur. For example, the epitope (NKILKVIRKNIVKK) that Mycograb targets shows high similarity between yeast, mice, and human homologues of Hsp90 (58), including A. fumigatus Hsp90 (NKIMKVIKKNIVKK, amino acids 383 to 396). Other abundant, fungus-specific surface proteins may represent better targets for immunotherapy as discussed in the following section.

To date, more than 100 antigens or immune-reactive proteins of A. fumigatus that react with sera from ABPA patients or animal models have been identified using classical immunobiological procedures (35–37). However, only a few recombinant allergens have been used commercially for diagnosis of allergic aspergillosis (5). Although the crystal structures of some allergens are known (61, 62), the issue of whether the allergen/protein exhibits special structural characteristics that are responsible for its allergenicity is still poorly understood. Thus, information is needed about the association of allergens with the different morphotypes (conidia, mycelium) and their exposed regions that may directly mediate the interaction with host components, such as IgE binding. Such knowledge creates the basis for understanding the immunological properties of protein antigens and is important for the establishment of new forms of diagnosis and treatment (63).

The cyclophilins, including Asp f 11 and Asp f 27 (62), are structurally conserved pan-allergens able to elicit IgE-mediated hypersensitivity reactions (62, 64, 65). It was reported previously that the conserved N81–N149 region of Rhizopus oryzae cyclophilin Rhi o 2 (see Fig. S5 in the supplemental material) is crucial for IgE recognition and cross-reactivity (64). In this study, however, the C-terminal region of Asp f 27, which is not conserved, was detected with biotinylation marks instead (Table 3; see also Fig. S5). This also provides a possible target for immunotherapy.

10.1128/mSphere.00535-20.5 FIG S5 Alignment of Asp f 27 and homologous proteins. Lysine residues of Asp f 27 detected with biotinylation marks in this study are indicated with arrows. Black squares indicate complete homology, and gray squares indicate changes in functionally conserved amino acids. Protein sequences of Asp f 27 (UniProtKB accession no. Q4WWX5), CaCYP1 (Candida albicans; UniProtKB accession no. P22011), and CPR1 (Saccharomyces cerevisiae; UniProtKB accession no. P14832), and Rhi o 2 (Rhizopus delemar; UniProtKB accession no. P0C1H7) were downloaded from UniProt Knowledgebase (www.uniprot.org). Protein sequences of Ole e 15 (Olea europaea; GenBank accession no. AVV30163.1), HuCYPA (Homo sapiens; GenBank accession no. AAH05982.1), and ROC5 (Arabidopsis thaliana; NCBI reference sequence accession no. NP_195213.1) were downloaded from NCBI (www.ncbi.nlm.nih.gov). Download FIG S5, EPS file, 1.2 MB. Copyright © 2020 Jia et al. 2020 Jia et al. This content is distributed under the terms of the Creative Commons Attribution 4.0 International license.

In addition to the cyclophilin allergens, several other allergens were detected with biotinylation sites on different morphotypes, such as the K50–K101 region of Asp f 6, the K304–K327 region of Asp f FDH, K238–K269, and the K303–K331 region of Asp f MDH (Table 3). Note that there were also regions of surface proteins that were detected on all morphotypes. For example, the K472–K486 region of Tef1, the K122 region of Afu5g10550, the K225–K231 region of Bgt2, and the K420–K435 region of the putative dihydrolipoamide dehydrogenase Afu2g02100 were all consistently surface exposed (Table 1). These peptides may represent promising candidate antigens for the development of monoclonal antibodies to be used for diagnosis and immunotherapy.

As published in our previous studies, the dynamic surfome of A. fumigatus is affected by various cultivation conditions, including the medium, temperature, and time, and contains many proteins secreted by nonclassical pathways (10, 11). Correspondingly, a recent study also revealed that the phenotypes of A. fumigatus germinating conidia vary among genetically identical conidia (66). Here, we detected 61, 69, and 85 proteins on dormant, swollen, and germinating conidia, respectively; while 136 proteins were found on the surface of hyphae (Fig. 3C). The comparison of the different surface proteomics data sets helped to identify the core surfome of A. fumigatus (Fig. 5). These proteins, which are consistently found on the surface of A. fumigatus, likely play a role in mediating the interaction of A. fumigatus with the host or other organisms.

## MATERIALS AND METHODS

### Fungal strains and cultivation.

All strains used in this study are listed in Table S2 in the supplemental material. A. fumigatus CEA10 was cultivated as described previously (11). Briefly, the CEA10 strain was inoculated on *Aspergillus* minimal medium agar plates with 1% (wt/vol) glucose for 7 days at 37°C. Conidia were harvested in sterile H_2_O and separated from hyphae and conidiophores by filtering (30-μm pore size; Miltenyi Biotec). For germination of swollen conidia, 1 × 10^10^ freshly collected conidia were incubated shaking in 50 ml RPMI 1640 (Lonza) for 5 h at 37°C. A total of 1 × 10^9^ conidia were germinated under the same set of conditions for 8 h to enrich for germlings and 1 × 10^9^ conidia in 100 ml RPMI 1640 for 14 h to enrich for hyphae.

### Biotinylation of surface proteins.

In previously published studies, Urban et al. incubated Candida albicans with Sulfo-NHS-LC-Biotin for 2 h at 4°C and de Miguel et al. incubated Trichomonas vaginalis with Sulfo-NHS-SS-biotin for 45 min on ice (25, 26). We chose a shorter time, since a longer incubation time or an incubation at elevated temperatures could increase the leakage of the biotinylation reagents inside the cell. Experiments were performed as described previously (26) with minor modification. Conidia, germlings, and hyphae were washed three times with PBS (pH 7.4) and then incubated in 5 ml of PBS containing 5 mg EZ-Link Sulfo-NHS-LC-Biotin (Thermo Fisher Scientific, catalog no. f21335) for 1 h at 4°C. As a nonbiotinylated control, fungal materials were also incubated in PBS alone. In all cases, the reaction was terminated by adding two volumes of 100 mM Tris-HCl (pH 7.4), and the reaction mixture was incubated for a further 30 min. The samples were then washed another three times with PBS (pH 7.4). All experiments were performed in three (dormant conidia, swollen conidia) or four biological (germlings, mycelia) replicates.

### Protein extraction and purification.

After addition of 1 ml of PBS (pH 7.4) containing protease inhibitor (Roche cOmplete; catalog no. f04693159001) (1 tablet per 10 ml) and 500 μl of 0.5-mm-diameter glass beads, conidia, germlings, and hyphae were disrupted using a FastPrep homogenizer with the following settings: 6.5 m/s, 3 times for 30 s each time. The samples were then centrifuged at 16,000 × *g* for 10 min at 4°C. The supernatants were collected and denoted “PBS extracts.” The pellets were washed twice with 1 M NaCl and another three times with 50 mM Tris-HCl (pH 7.4) followed by extraction with SDS buffer (2% [wt/vol] SDS, 50 mM Tris-HCl [pH 7.4], 100 mM EDTA, 150 mM NaCl, 40 mM dithiothreitol [DTT]) for 10 min at 100°C. For purification of the biotinylated proteins, a 1-mg volume of ROTI-MagBeads streptavidin (Carl Roth, catalog no. fHP57.1) was equilibrated by three washes in PBS (pH 7.4) and then incubated with the biotinylated or nonbiotinylated protein samples for 2 h in a rotating mixer at 4°C. The beads were washed five times with four different urea buffers (buffer A, consisting of 8 M urea, 200 mM NaCl, 2% [wt/vol] SDS, and 100 mM Tris [pH 8]; buffer B, consisting of 8 M urea, 1.2 M NaCl, 0.2% [wt/vol] SDS, 100 mM Tris [pH 8], 10% [wt/vol] ethanol, and 10% [wt/vol] isopropanol; buffer C, consisting of 8 M urea, 200 mM NaCl, 0.2% [wt/vol] SDS, 100 mM Tris [pH 8], 10% [vol/vol] ethanol, and 10% [vol/vol] isopropanol; or buffer D, consisting of 8 M urea, 100 mM Tris [pH 8]) as previously described (26). Bound proteins were isolated by incubating the streptavidin beads with 170 μl of elution buffer (30 mM d-biotin, 8 M urea, 2% [wt/vol] SDS, 100 mM Tris [pH 8]) for 30 min at 50°C.

### Western blot analysis.

Proteins were separated by SDS-PAGE using NuPAGE 4%-to-12% (wt/vol) Bis-Tris gradient gels (Thermo Fisher Scientific) and were then transferred onto a polyvinylidene difluoride (PVDF) membrane using an iBlot 2 dry blotting system (Thermo Fisher Scientific). To detect biotinylated proteins, 5 μg of total protein and 20 μl of purified protein were loaded and membranes were blocked with Western blocking reagent (Roche) and then incubated with Pierce streptavidin-horseradish peroxidase (HRP) (Thermo Fisher Scientific, catalog no. f21130) (1:2,000) overnight at 4°C. To detect the Myc-tagged proteins, a 10-μg volume of total protein was loaded and membranes were blocked with a 5% (wt/vol) solution of skim milk and then incubated with primary antibody (Myc-tagged mouse monoclonal antibody [MAb]; Cell Signaling Technology, catalog no. f2276) (1:1,000) overnight at 4°C. Hybridization with a secondary antibody (HRP-linked anti-mouse IgG; Cell Signaling Technology, catalog no. f7076) was performed for 1 h at room temperature. Chemiluminescence of HRP substrate was detected with a Fusion FX7 system (Vilber Lourmat, Germany).

### Immunofluorescence microscopy.

The fungal materials were washed three times with PBS (pH 7.4) and then blocked with 1% (wt/vol) bovine serum albumin (BSA)–PBS for 1 h at room temperature. For detection of surface biotinylation, A. fumigatus conidia, germlings, and hyphae were incubated with Alexa Fluor 635-conjugated streptavidin (Thermo Fisher Scientific, catalog no. S32364) (1:100) for 1 h in the dark. The surface localization of Myc-tagged proteins was examined using an anti-Myc primary antibody (Myc-tagged mouse MAb; Cell Signaling Technology, catalog no. f2276) (1:100) for 2 h at room temperature and a secondary antibody (donkey anti-mouse IgG H&L Alexa Fluor 568, Abcam catalog no. fab175472) (1:500) for 1 h at room temperature in the dark. After three washes with PBS, the samples were examined under a Zeiss Axio Imager M2 microscope.

### In-solution protein digestion with trypsin.

Eluted protein samples (150 μl) were reduced by adding 4 μl of 500 mM TCEP [Tris(2-carboxyethyl)phosphine]–100 mM TEAB (triethylammonium bicarbonate) for 1 h at 55°C. A 4-μl volume of 625 mM iodoacetamide was added to each sample followed by incubation for 30 min at room temperature in the dark. Proteins were then precipitated using the methanol-chloroform-water method (67). Protein samples were resolubilized in 100 μl of 100 mM TEAB and sonicated for 10 min. The protein content was measured with a Merck Millipore Direct Detect infrared spectrometer. The samples were treated with trypsin/Lys-C protease mix (Promega, catalog no. fV5072) at a protease/protein ratio of 1:25 for 16 h at 37°C. The reaction was stopped with 10 μl of 10% (vol/vol) formic acid, and the reaction mixture was subjected to evaporation using a SpeedVac (Thermo Fisher Scientific). Peptides were resolubilized in 25 μl 0.05% (vol/vol) trifluoroacetic acid (TFA)–2% (vol/vol) acetonitrile (ACN)–water and sonicated for 15 min in a water bath before transfer to a 10-kDa-molecular-weight cutoff filter. After 15 min of centrifugation at 14,000 × *g* (4°C), the samples were transferred to high-performance liquid chromatography (HPLC) vials and stored at –20°C.

### Genetic manipulation of A. fumigatus.

All oligonucleotides utilized in this study are listed in Table S3. For surface localization studies, a pLJ*-Ssc70-Myc* construct was generated using plasmid pTH1 (68) as the backbone. A 3,276-bp DNA fragment containing a 1,010-bp 5′ promoter region, the *ssc70* gene without TAA, and an in-frame fused Myc-tagged coding sequence was PCR amplified from genomic DNA using primers Ssc70-M_F and Ssc70-M_R. Another 371-bp DNA fragment was amplified from plasmid pTH1 using primers Myc_F and pTH_R. These two fragments were mixed as the template and amplified using primers Ssc70-M_F and pTH_R to generate the *Ssc70*-*Myc* fragment. *Ssc70*-*Myc* fragment was digested with KpnI and NotI and then inserted into the pTH1 plasmid using T4 ligase (Thermo Fisher Scientific) to yield the pLJ*-Ssc70-Myc* plasmid. The pLJ*-Ssc70-Myc* plasmid was then used as the backbone to generate other Myc-tagged constructs. For example, a 3,073-bp DNA fragment containing the *hsp70* gene and the promoter region was PCR amplified from genomic DNA using primers Hsp70-M_F and Hsp70-M_R. The DNA fragment was then inserted into the KpnI- and HindIII-digested pLJ*-Ssc70-Myc* backbone using a CloneEZ PCR cloning kit (GenScript) to get plasmid pLJ*-Hsp70-Myc*. Primers BipA-M_F and BipA-M_R were used to amplify the *bipA* gene with a promoter region. Primers Ssz-M_F and Ssz-M_R were used to amplify the *ssz* gene with a promoter region. The plasmids were used to transform protoplasts from A. fumigatus strain A1160 (CEA17 Δ*akuB*^KU80^) (69).

### LC-MS/MS analysis.

LC-MS/MS analysis of tryptic peptides was performed on an Ultimate 3000 RSLC nano instrument coupled to a Q Exactive HF mass spectrometer (both Thermo Fisher Scientific) in two analytical replicates. Tryptic peptides were trapped for 4 min on an Acclaim Pep Map 100 column (2 cm by 75 μm, 3-μm pore size) at a flow rate of 5 μl/min. The peptides were then separated on an Acclaim Pep Map column (50 cm by 75 μm, 2-μm pore size) using a binary gradient (A, 0.1% [vol/vol] formic acid–H_2_O; B, 0.1% [vol/vol] formic acid–90:10 [vol/vol] ACN/H_2_O) as follows: 0 min at 4% B, 6 min at 8% B, 30 min at 12% B, 75 min at 30% B, 85 min at 50% B, 90 to 95 min at 96% B, and 95.1 to 120 min at 4% B. Positively charged ions were generated by the use of a Nanospray Flex ion source (Thermo Fisher Scientific) using a stainless steel emitter with 2.2-kV spray voltage. Ions were measured in data-dependent MS2 (Top15) mode. Precursor ions (z = 2 to 5) were scanned at *m*/*z* 300 to 1,500 (resolution [R], 120,000 full width at half maximum [FWHM]; automatic gain control [AGC] target, 3·10^6^; maximum injection time [IT_max_], 120 ms). Fragment ions generated in the higher-energy collisional dissociation (HCD) cell at 30% normalized collision energy using N_2_ were scanned (R, 15,000 FWHM; AGC target, 2·10^5^; IT_max_, 90 ms) using a dynamic exclusion duration of 30 s.

### Database search and data analysis of trypsin-cleaved surface peptides.

The MS/MS data were searched against the Aspergillus Genome database (AspGD) entries for Aspergillus fumigatus Af293 (http://www.aspergillusgenome.org/download/sequence/A_fumigatus_Af293/current/; 3 February 2019) using Proteome Discoverer (PD) 2.2 and the algorithms of Mascot 2.4.1, Sequest HT, and MS Amanda 2.0. Two missed cleavages were allowed for tryptic peptides. The precursor mass tolerance was set to 10 ppm, and the fragment mass tolerance was set to 0.02 Da. Dynamic modifications were set as oxidation (+15.995 Da) of Met and NHS-LC-Biotin (+339.162 Da) modification of Lys and the protein N terminus. The static modification was set to carbamidomethylation (+57.021 Da) of Cys. One unique rank 1 peptide with a strict target false-discovery (FDR) rate of <1% on both the peptide and protein levels (compared against a reverse-decoy database) was required for positive protein hits. Only proteins identified in at least two different biological replicates were considered. Protein abundance quantification was performed by the use of the Minora algorithm of PD2.2 (area under the curve approach).

### Data availability.

The mass spectrometry proteomics data have been deposited to the ProteomeXchange Consortium via the PRIDE (70) partner repository with the data set identifier PXD018071.
