# Supplementary figures and images for: Biotinylated Surfome Profiling Identifies Potential Biomarkers for Diagnosis and Therapy of Aspergillus fumigatus Infection
Source: mSphere. 2020 Aug 12;5(4):e00535-20. doi: 10.1128/mSphere.00535-20 (PMC7426169; doi:10.1128/mSphere.00535-20)

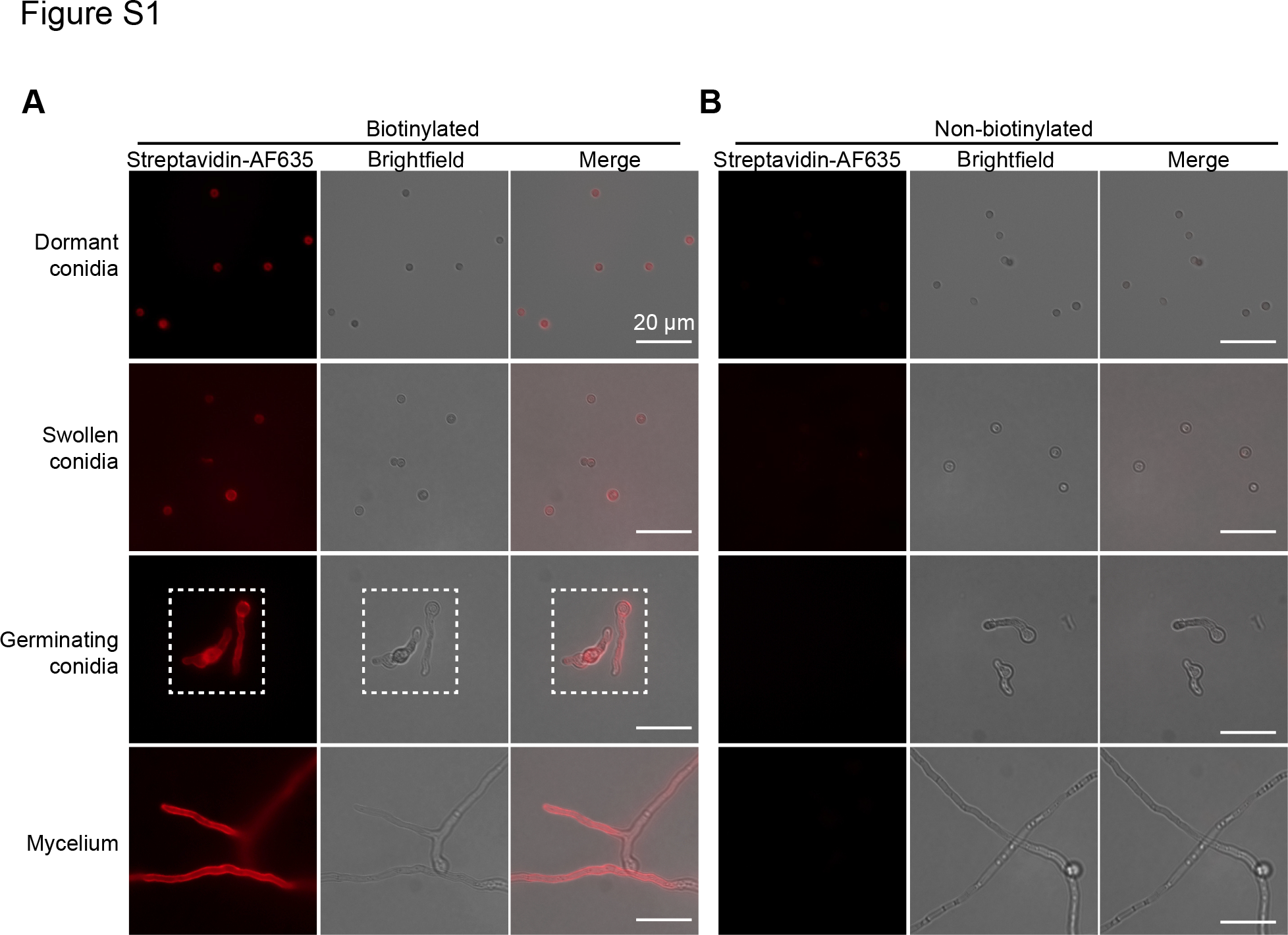

Supplement: FIG S1 [file mSphere.00535-20-sf001.tif]

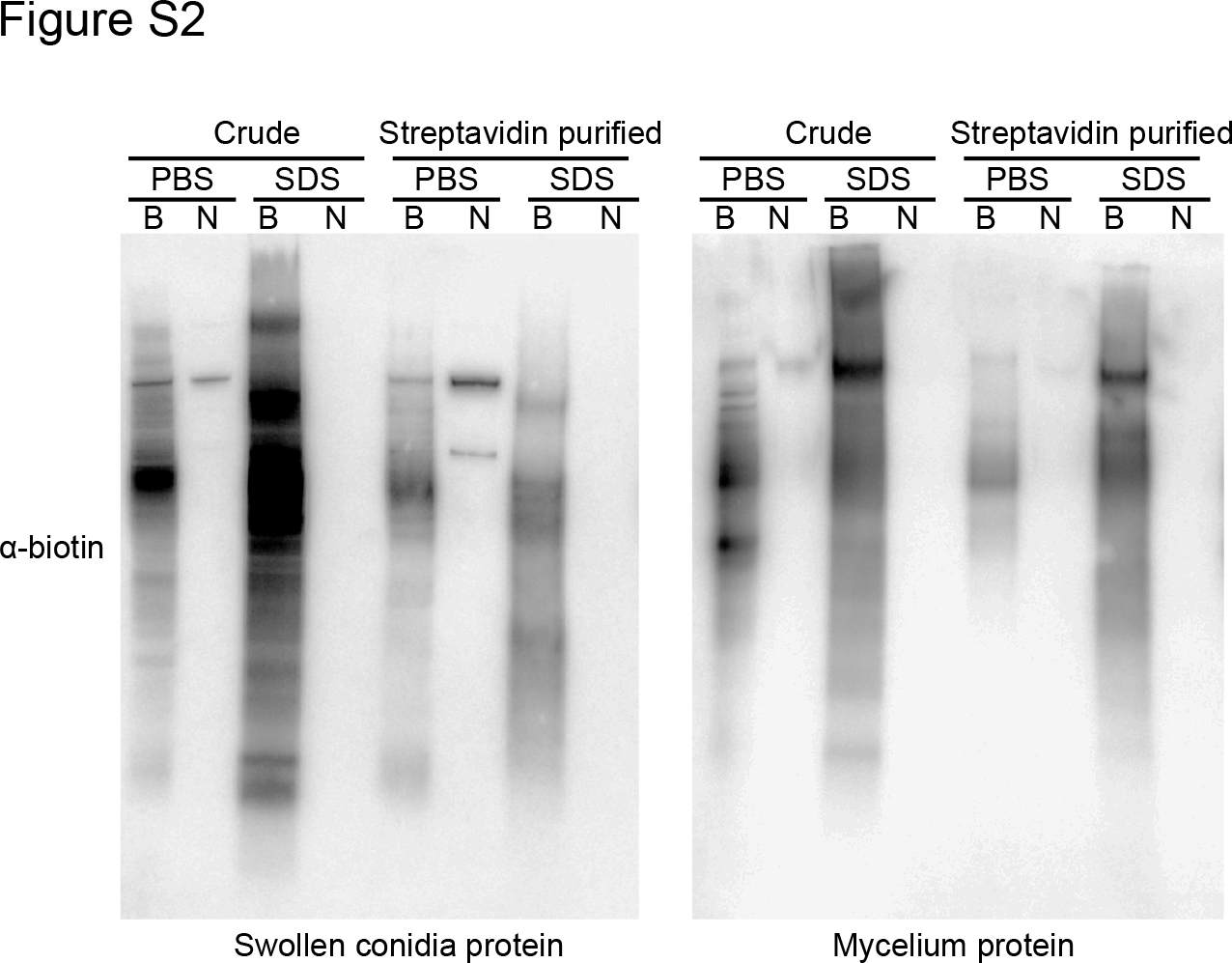

Supplement: FIG S2 [file mSphere.00535-20-sf002.tif]

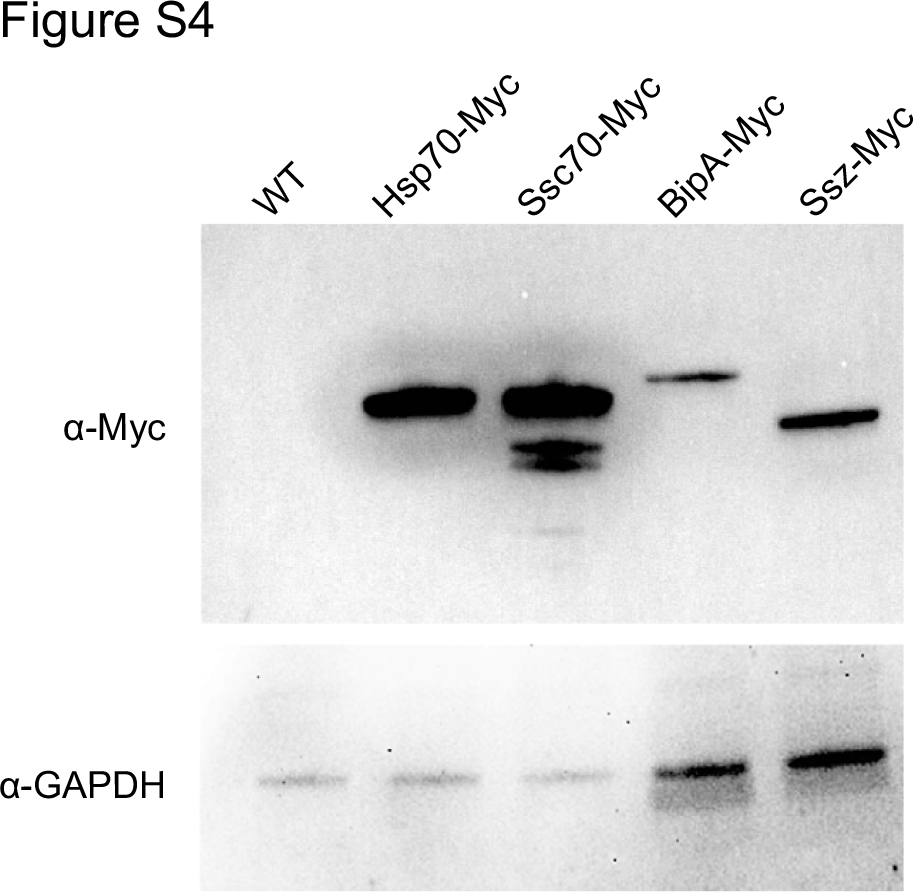

Supplement: FIG S4 [file mSphere.00535-20-sf004.tif]
