# Supplementary material for: Biotinylated Surfome Profiling Identifies Potential Biomarkers for Diagnosis and Therapy of Aspergillus fumigatus Infection
Source: mSphere. 2020 Aug 12;5(4):e00535-20. doi: 10.1128/mSphere.00535-20 (PMC7426169; doi:10.1128/mSphere.00535-20)
Supplement: TABLE S2 [file mSphere.00535-20-st002.docx]

**Table S2**. *A. fumigatus* strains used in this study.

| **Description** | **Relevant genotype** | **Reference or source** |
| --- | --- | --- |
| Wild type | CEA10 | Fungal Genetics Stock Center (A1163) |
| A1160 | Δ*ku80* pyrG^+^ | da Silva Ferreira et al., 2006 (69) |
| *hsp70-Myc* | A1160 *hsp70*-*Myc*-*ptrA*; PT^R^ | This study |
| *ssc70-Myc* | A1160 *ssc70*-*Myc*-*ptrA*; PT^R^ | This study |
| *bipA-Myc* | A1160 *bipA*-*Myc*-*ptrA*; PT^R^ | This study |
| *ssz-Myc* | A1160 *ssz*-*Myc*-*ptrA*; PT^R^ | This study |
