# Supplementary material for: Biotinylated Surfome Profiling Identifies Potential Biomarkers for Diagnosis and Therapy of Aspergillus fumigatus Infection
Source: mSphere. 2020 Aug 12;5(4):e00535-20. doi: 10.1128/mSphere.00535-20 (PMC7426169; doi:10.1128/mSphere.00535-20)
Supplement: TABLE S3 [file mSphere.00535-20-st003.docx]

**Table S3**. Oligonucleotides used in this study.

| **Name** | **Sequence 5' to 3'** | **Usage** |
| --- | --- | --- |
| Ssc70-M_F | TTCGAGCTCGGTACCTTCTCGGAATGACATTAAGC | Plasmid pLJ-Ssc70-Myc |
| Ssc70-M_R | AAGATCCTCCTCGGAGATAAGCTTCTGCTCGGGCTTGTTCTCACCCTG |  |
| Myc_F | ATCTCCGAGGAGGATCTTGGCTCCGGCTCCTAAAGCGGCCGCCCGGCTGC |  |
| pTH_R | CCTGAGTGGCCATCGAATTC |  |
| Hsp70-M_F | TTCGAGCTCGGTACCTGAGAGAGCCGAGGGCTACG | Plasmid pLJ-Hsp70-Myc |
| Hsp70-M_R | CCTCCTCGGAGATAAGCTTCTGCTCGTCAAGCTCCTCAGGGCGCT |  |
| BipA-M_F | TTCGAGCTCGGTACCCCCGAGTACATTGATAGAGG | Plasmid pLJ-BipA-Myc |
| BipA-M_R | CTCGGAGATAAGCTTCTGCTCCAGTTCGTCATGTCCGCTGG |  |
| Ssz-M_F | TTCGAGCTCGGTACCCCTCCGTTCAATGGGCCCAA | Plasmid pLJ-Ssz-Myc |
| Ssz-M_R | CTCGGAGATAAGCTTCTGCTCAGCCTTGGGCGACTCGACGG |  |
